# Everolimus plus exemestane versus bevacizumab-based chemotherapy for second-line treatment of hormone receptor-positive metastatic breast cancer in Greece: An economic evaluation study

**DOI:** 10.1186/s12913-015-0971-4

**Published:** 2015-08-05

**Authors:** Georgia Kourlaba, Vasiliki Rapti, Athanasios Alexopoulos, John Relakis, Georgios Koumakis, Magdalini Chatzikou, Nikos Maniadakis, Vassilis Georgoulias

**Affiliations:** The Stavros Niarchos Foundation-Collaborative Center for Clinical Epidemiology and Outcomes Research (CLEO), National and Kapodistrian University of Athens, School of Medicine, Athens, Greece; HYGEIA Hospital, Maroussi, Greece; Department of Health Services Organization & Management, National School of Public Health, Athens, Greece; 2nd Department of Pathology-Oncology, St. Savvas Hospital, Athens, Greece; Novartis Hellas SA, Metamorphosis, Metamorfossi, Greece; Department of Medical Oncology, University General Hospital of Heraklion, Heraklion, Greece

**Keywords:** Metastatic breast cancer, Estrogen receptor positive, Cost-effectiveness, Everolimus, Bevacizumab

## Abstract

**Background:**

The objective of our study was to conduct a cost-effectiveness (CE) study of combined everolimus (EVE) and exemestane (EXE) versus the common clinical practice in Greece for the treatment of postmenopausal women with HR+/HER2- advanced breast cancer (BC) progressing on nonsteroidal aromatase inhibitors (NSAI). The combinations of bevacizumab (BEV) plus paclitaxel (PACL) and BEV plus capecitabine (CAPE) were selected as comparators.

**Method:**

A Markov model, consisting of three health states, was used to describe disease progression and evaluate the CE of the comparators from a third-party payer perspective over a lifetime horizon. Efficacy and safety data as well as utility values considered in the model were extracted from the relevant randomized Phase III clinical trials and other published studies. Direct medical costs referring to the year 2014 were incorporated in the model. A probabilistic sensitivity analysis was conducted to account for uncertainty and variation in the parameters of the model. Primary outcomes were patient survival (life-years), quality-adjusted life years (QALYs), total direct costs and incremental cost-effectiveness ratios (ICER).

**Results:**

The discounted quality-adjusted survival of patients treated with EVE plus EXE was greater by 0.035 and 0.004 QALYs, compared to BEV plus PACL and BEV plus CAPE, respectively. EVE plus EXE was the least costly treatment in terms of drug acquisition, administration, and concomitant medications. The total lifetime cost per patient was estimated at €55,022, €67,980, and €62,822 for EVE plus EXE, BEV plus PACL, and BEV plus CAPE, respectively. The probabilistic analysis confirmed the deterministic results.

**Conclusion:**

Our results suggest that EVE plus EXE may be a dominant alternative relative to BEV plus PACL and BEV plus CAPE for the treatment of HR+/HER2- advanced BC patients failing initial therapy with NSAIs.

**Electronic supplementary material:**

The online version of this article (doi:10.1186/s12913-015-0971-4) contains supplementary material, which is available to authorized users.

## Background

Breast cancer (BC) is the most prevalent neoplasm, accounting for 5.2 million cases worldwide [1]. While being the fifth leading cause of cancer-related deaths, it is still the most frequent cause of cancer death for women in both developing and developed regions [2]. It is estimated that about 5 % to 10 % of BCs are metastatic at diagnosis [3]. Moreover, despite the continuing advances in therapy, approximately 20 % to 30 % of early BC cases will eventually become metastatic [4, 5]. In this advanced stage the cancer can no longer be cured but it can be controlled for several years.

The disease is associated with a substantial economic burden due to increased resource utilization. From diagnosis to death, the total cost for the management of patients with metastatic BC (mBC) has been reported to range from $41,590 to $82,973 (adjusted to 2005 US dollars) [6–9]. Outpatient services have been found to account for 29 % of total cost (driven by diagnostic imaging and radiation therapy), followed by medication other than chemotherapy (26 %), chemotherapy (25 %), and inpatient care (20 %) [10]. Interestingly, Medicare data have revealed that the direct cost is lower in older mBC patients compared to younger patients, implying that the cost of illness is inversely proportional to age [9]. Furthermore, productivity loss and other indirect costs are substantially higher in mBC patients than in early BC patients or the general population, underscoring the economic burden of mBC [11].

The treatment of mBC usually involves hormone therapy and/or chemotherapy, with or without monoclonal antibodies (i.e. bevacizumab [BEV], trastuzumab [TRZ]). The cytological and histological documentation of the disease prior to treatment is essential in order to select the most effective therapy. In this context, the status and level of hormone receptors (HR) – estrogen receptors (ER) and progesterone receptors (PR) – as well as the positivity of human epidermal growth factor receptor 2 (HER2/neu) at the time of recurrence should be considered [12].

In cases of postmenopausal women with HR+ advanced BC, aromatase inhibitors (steroidal or nonsteroidal) are indicated as the standard initial treatment. Nonetheless, the majority of patients either do not respond to the initial treatment or develop resistance [13]. Alternative treatment options, such as ER antagonists (e.g. tamoxifen [ΤΑΜ]) and ER down-regulators (e.g. fulvestrant [FULV]), are considered of limited efficacy within the frame of endocrine resistance that occurs after first-line treatment with aromatase inhibitors [13, 14]. In July 2012, the European Commission approved the addition of everolimus (EVE) to the aromatase inhibitor exemestane (EXE) for the treatment of post-menopausal women with HR+/HER2- advanced BC progressing on nonsteroidal aromatase inhibitors (NSAI: i.e. anastrozole, letrozole). EVE is a new treatment option that inhibits the hyper-activation of the mammalian target of rapamycin, a protein that is associated with BC progression and the development of endocrine resistance [15]. The clinical benefit of the EVE plus EXE combination, regardless of ethnicity, was established through the Phase III BOLERO-2 trial (Breast cancer trials of OraL EveROlimus-2) [16, 17].

BC patients whose tumors have progressed on hormone therapy are candidates for chemotherapy [12]. Single agents, such as paclitaxel (PACL) and capecitabine (CAPE), have shown efficacy in mBC [18–23]. Nonetheless, the addition of BEV – a monoclonal antibody directed against all isoforms of vascular endothelial growth factor-A – in PACL or CAPE is common practice and has been evaluated in clinical trials [24, 25].

Although the addition of EVE to hormone therapy and BEV to chemotherapy seems to be more efficacious than the corresponding monotherapies, these combinations may impose additional costs on third-party payers. However, the recent climate of the major financial crisis, especially in Greece, has resulted in strong budgetary constraints. This fact makes imperative the use of treatments not only clinically effective but also economically efficient, to maximize the value, in other words the benefit, of the money spent on health care. This need has led to the use of economic assessment models to evaluate the technologies employed in health care systems.

Hence, the objective of the present analysis was to evaluate the cost-effectiveness of EVE combined with EXE versus the common clinical practice in Greece for the treatment of postmenopausal women with HR+/HER2- advanced BC progressing on NSAI.

## Methods

A Markov model was adapted locally to reflect the natural progression of postmenopausal women with ER+/HER2-. The model evaluated the cost-effectiveness of EVE plus EXE versus BEV plus PACL and BEV plus CAPE over a lifetime horizon. It should be noted that, although other hormonal therapies or chemotherapies as monotherapies could be considered as comparators in the present analysis, they were not selected since they do not constitute the common clinical practice in Greece, probably because of their limited efficacy. The analysis was performed from a payer’s perspective (EOPYY). Costs and outcomes that occurred beyond one year were discounted at a 3.5 % annual rate, which is the standard practice in Greece as well as other jurisdictions. No approval by an appropriate ethical committee was required as no humans were involved in this study.

### Model structure

**Fig. 1:**
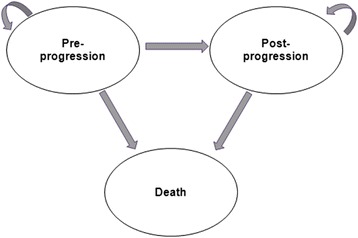
Markov model structure

The Markov model consists of three mutually exclusive health states: pre-progression, post-progression, and death as an absorbing state (Fig. 1). Patients entered the model at the pre-progression health state and were treated with one of the model comparators until disease progression or death. When patients moved to the post-progression health state they either remained or transitioned to death. The cycle length of the model was one month.

### Target population

The hypothetical cohort of patients entering the Markov model was assumed to have similar baseline characteristics to the population included in the Phase III BOLERO-2 randomized clinical trial [16].

### Efficacy

The efficacy of treatment in the pre-progression state of the model was quantified in terms of progression-free survival (PFS) and overall survival (OS). In particular, in the base case scenario, the efficacy of EVE plus EXE was derived from the BOLERO-2 trial data using local radiological assessment, whereas data obtained from central radiological assessment were used in a sensitivity analysis [16, 17]. Due to the “lifetime” character of the analysis, OS and PFS were extrapolated beyond the follow-up period of the BOLERO-2 trial through the application of parametric survival curves. The parametric survival curves were estimated using four distributions: exponential-endpoint, Weibull, exponential-curve, and log-logistic. Based on the statistical properties of the curve-fitting analysis, the Weibull distribution was selected as the best-fit curve for OS and PFS in the base-case analysis. The other parametric distributions were used in the sensitivity analyses.

**Fig. 2:**
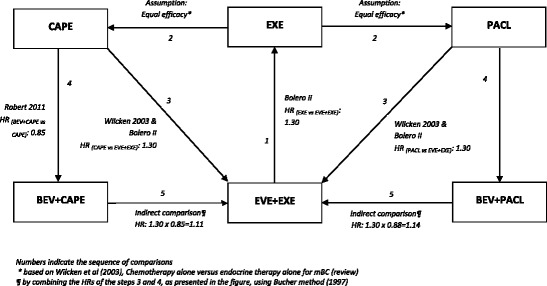
Assumptions and indirect comparisons to obtain estimations for the overall survival of comparators

**Fig. 3:**
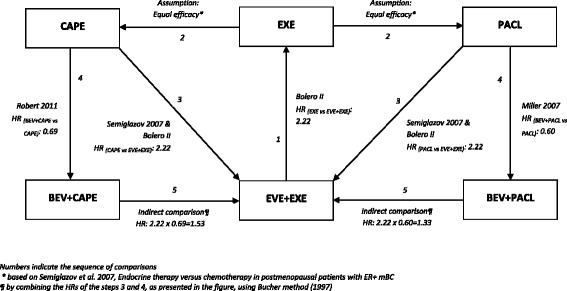
Assumptions and indirect comparisons to obtain estimations for the progression free survival of comparators

To estimate the PFS and OS for the comparators, relevant hazard ratios (HRs) were applied to the OS and PFS functions of EVE plus EXE. To be more precise, in the absence of head-to-head clinical trials between EVE plus EXE and the selected comparators (BEV plus PACL and BEV plus CAPE), indirect methods and assumptions, similar to those considered in the model submitted to the National Institute for Clinical Excellence (NICE) [15], were used to obtain estimates for HRs of the comparators against EVE plus EXE. The assumptions and indirect comparisons employed to calculate the corresponding HRs for OS and PFS are depicted in Figs. 2 and 3.

Finally, it was assumed that the post-progression treatment sequences did not affect patients’ survival or quality of life (i.e. utilities). This assumption is considered reasonable, since the effect of post-progression treatment is indirectly modeled through the OS related to the pre-progression treatment. To this end, identical post-progression treatment sequences were considered for the comparators and were only allowed to affect the related costs that arose.

### Comparator dosing and administration

The treatment doses for each comparator matched those used in Phase III clinical trials, so that dosing could be correlated with clinical efficacy (Additional file 1: Appendix I). Moreover, patients were assumed to continue treatment until progression or death. Nonetheless, the relative dose intensity was also incorporated in the model, allowing for the fact that some patients may not take the planned dose for the whole treatment period until progression or death. The relative dose intensities of EVE and EXE (when these are coadministered) were derived from the BOLERO-2 trial (86 % and 100 %, respectively). In view of the lack of data, the relative dose intensity for the other comparators was assumed to be equal to that of EVE in the BOLERO-2 trial (86 %)[17].

### Safety

In accordance with usual practice, Grade III/IV adverse events were considered in the model. Among the adverse events that may be experienced by mBC patients treated with one of our comparators, only those requiring substantial use of health care resources were considered in the analysis. Based on local expert opinion, the following adverse events were considered: nausea, vomiting, pneumonitis, allergy, anemia, neutropenia, arthralgia and myalgia. The frequency of adverse events was extracted from relevant clinical trials [16, 24, 25].

### Utilities

Utilities in the Markov model were applied to the health state and were not treatment-related. The pre-progressed and the post-progressed utility values were derived from the study of Lloyd et al. [26], as used in several technology appraisals for mBC submitted to NICE (i.e. FULV, eribulin, lapatinib and trastuzumab) [15]. Calculations were based on a sample of the general public in the UK (using the Standard Gamble technique) and since the people valuing the health states were younger than the patients in the BOLERO-2 trial (40.16 as opposed to 62.1), age-adjusted estimations of utilities were eventually incorporated in the model (0.7733 for the pre-progression state and 0.4964 for the post-progression state).

### Costing methods

Since the analysis was conducted from the third-party payer perspective, only direct medical costs reimbursed by EOPYY were considered in the model (i.e. hospitalization, physician visits, pre-treatment medications, complementary or prophylactic treatments, imaging tests, lab tests, and costs for the management of adverse events). The resource utilization associated with each health state was based on local expert opinion. The volumes of resource units were combined with the corresponding local unit costs to aggregate a total cost per health state. All costs applied in the analysis refer to the year 2013.

#### Pre-progression state costs

Costs in the pre-progression health state comprised acquisition and administration costs for the comparators and pretreatment medications, as well as costs related to the disease and adverse events management (i.e. imaging and lab tests, complementary treatments). Information about the pretreatment medication, as well as the resource utilization related to disease and adverse events management, were obtained from a local expert to reflect the common clinical practice in Greece. In brief, patients following chemotherapy regimens were considered to be receiving pretreatment medication and prophylactic treatment for neutropenia and anemia, as opposed to patients treated with EVE plus EXE. Moreover, disease management in the pre-progression state was considered to include laboratory and imaging tests. Details of the resource utilization are presented in Additional file 1: *Appendix I*.

##### Drug acquisition and administration costs

The drug acquisition costs were calculated by combining the drug dosing schedules with the corresponding reimbursed drug prices. In Greece, the reimbursed drug prices depend on the way each drug is provided in the health care system (hospital, EOPYY pharmacies, retail pharmacies) as well as the way these medicines are administered (i.e. intravenously, orally). In this context, the reimbursed price was calculated as the hospital price plus a mark-up of 5 % for BEV and PACL, as the hospital price minus a rebate of 5 % for EVE, CAPE and other high cost drugs delivered in the outpatient setting, while for drugs dispensed in community pharmacies (such as EXE), the reimbursed prices were calculated based on the social security reimbursement prices and retail prices. Hospital and retail prices were obtained from the Price Bulletin published in August 2013 and social security reimbursement prices were extracted from the Positive List published in October 2013.

The costs for disease management of patients in the pre-progression state as well as the management of adverse events were calculated by multiplying the number of resource units (obtained from a local expert) with the corresponding costs per unit (i.e. reimbursed costs, assuming that all patients were treated in the public sector). The costs related to adverse event management were one-off costs. All unit costs are presented in Additional file 2: *Appendix II*, while the total pre-progressed cost per cycle is presented in Table 1.

**Table 1:** Cost inputs per cycle per model health state

	EVE plus EXE	BEV plus PACL	BEV plus CAPE
Total Pre-progression cost/cycle			
*Drug acquisition cost*	2,447.75 €	3,806.42 €	3,510.97 €
*Administration cost*	0 €	260.89 €	115.95 €
*Pre-treatment costs*	0 €	82.58 €	36.69 €
*Lab tests*	42.82 €	52.41 €	48.25 €
*Monitoring cost*	52.34 €	52.34 €	52.34 €
*Prophylactic treatment cost*	-	274.72 €	416.77 €
Total post-progression cost/cycle	1,057.41 €	1,057.41 €	1,057.41 €
End-of-life cost	823.60 €	823.60 €	823.60 €

*EVE: everolimus; EXE: exemestane; BEV: bevacizumab; PACL: paclitaxel; CAPE: capecitabine*

#### Post-progression state costs

In the post-progression state, for all comparators, it was assumed that patients were assigned to one of two mutually exclusive treatment strategies, based on a local expert’s opinion. To be more precise, 50 % of patients experiencing disease progression, regardless of the treatment received in the pre-progression state, were considered to be treated with a 3rd line hormonal therapy (i.e. FULV), a 4th line chemotherapy (i.e. docetaxel [DOC]) and then with supportive palliative care (lonarid/fentanyl patches), whereas the remaining 50 % of patients received chemotherapy as 3rd (CAPE plus Vinorelbine) and 4th line treatment (DOC) and then supportive palliative care. Additional file 3: *Appendix III* shows in detail the resources employed during treatment, dosing schedules, average hospitalization and monitoring requirements .

In order to calculate the average cost per month in the post-progression state, the monthly cost was calculated for each treatment line (3^rd^ and 4^th^) in the two alternative strategies (drug costs, monitoring costs and hospitalization costs). Subsequently, for each strategy, the monthly cost per treatment line was weighted based on its duration (i.e. 12 months, 6 months) to obtain a total treatment strategy cost. Finally, based on the patients’ allocation to these strategies, as indicated by the medical expert (50 %-50 %), the average cost per month in the post-progression state was calculated.

The drug acquisition costs as well as the monitoring costs were calculated as described in the pre-progression state. The total post-progressed cost per cycle used in the analysis is presented in Table 1.

### Data analysis

The cost-effectiveness of EVE plus EXE over the comparators BEV plus PACL and BEV plus CAPE was evaluated by calculating the incremental cost-effectiveness ratio (ICER). For a treatment to be considered cost-effective, a willingness-to-pay (WTP) threshold of €36,000 per quality-adjusted life year (QALY) gained was used in the current analysis. This is based on the WHO guidelines, which state that a treatment should be considered cost-effective if the ICER is between 1 and 3 times the GDP per capita of that country and a treatment is considered highly cost effective at less than 1 times the GDP per capita [27]. The GDP per capita in Greece was estimated at €17,000, taken from the IMF estimation of GDP per capita using current prices [28].

Sensitivity analyses were undertaken to test the robustness of the results, by varying either individual parameters between low and high values within plausible ranges or the structural assumptions adopted in the model. However, the majority of parameters used in the current model are subject to variation. Therefore, in order to deal with uncertainty, a probabilistic sensitivity analysis (PSA) was performed using a Monte Carlo simulation. In this analysis, probability distribution was assigned around each parameter (i.e. costs, utilities, etc.) and cost-effectiveness results associated with simultaneously selecting random values from those distributions were generated. In particular, utility values are restricted to the interval zero to one, and hence they were varied according to a beta distribution. The gamma distribution and the lognormal distribution were applied for the cost and effectiveness variables, respectively.

One thousand estimates of costs, QALYs, and incremental cost per QALY gained were then obtained by performing the bootstrapping technique. A cost-effectiveness acceptability curve (CEAC) was plotted, showing the proportion of simulations that are considered cost-effective at different levels of willingness to pay per QALY gained.

## Results

### Deterministic results

The Markov model predicted that the discounted quality-adjusted survival of patients treated with EVE plus EXE would be greater compared to those treated with BEV plus PACL and BEV plus CAPE, by 0.035 and 0.004 QALYs, respectively. Moreover, the total lifetime cost per patient for EVE plus EXE, BEV plus PACL, and BEV plus CAPE was estimated to be €55,022, €67,980, and €62,822, respectively. Hence, the use of EVE plus EXE may result in a cost saving of €12,958 over BEV plus PACL and €7,800 over BEV plus CAPE. The observed difference in the total lifetime cost between EVE plus EXE and BEV plus PACL was mainly attributable to the drug acquisition and administration cost (EVE plus EXE: €25,727 vs. BEV plus PACL: €32,960), since BEV and PACL, apart from being a more expensive treatment combination, was associated with higher administration costs. Furthermore, BEV plus PACL was associated with a significantly higher pre-progression cost (EVE plus EXE: €1,000 vs. BEV plus PACL: €3,744), which may be attributed to the pre-treatment medications as well as the prophylactic treatments required. The higher pre-progression cost of the BEV plus CAPE arm (€3,946) accounted for the difference in the total lifetime cost between EVE plus EXE and BEV plus CAPE, while the BEV plus CAPE alternative generated an incremental cost of €4,813 in the post-progression state (EVE plus EXE: €27,495 vs. BEV plus CAPE: €32,308) (Table 2).

Based on the above, EVE plus EXE seems to be a dominant alternative over BEV plus PACL and BEV plus CAPE in a lifetime horizon, as the first combination is associated with a greater health benefit and a lower total lifetime cost (Table 2).

**Table 2:** Base case results for EVE plus EXE vs. BEV plus PACL and BEV plus CAPE for the treatment of ER+ mBC patients

				Incremental analysis	
Outcomes	EVE plus EXE	BEV plus PACL	BEV plus CAPE	BEV plus PACL	BEV plus CAPE
Treatment and administration costs*	25,727 €	32,960 €	25,832 €	−7,233 €	−105 €
AE costs (grade 3/4)*	62 €	5 €	1 €	57 €	61 €
Pre-progression background costs*	1,000 €	3,744 €	3,946 €	−2,744 €	−2,946 €
Post-progression background costs*	27,495 €	30,534 €	32,308 €	−3,039 €	−4,813€
Terminal care costs*	737.51 €	736.41 €	734,77 €	1.10 €	−2.74 €
Total costs*	55,022 €	67,980 €	62,822 €	−12,958 €	−7,800 €
QALYS*: Pre-progressed	0.648	0.494	0.456	0.154	0.192
QALYS*: Post-progressed	1.076	1.195	1,264	−0.119	−0.188
Total QALYS*	1.724	1.689	1,720	0.035	0.004
Life years†: Pre-progressed	0.899	0.689	0.604	0.210	0.295
Life years†: Post-progressed	2.422	2.679	2.833	−0.257	−0.411
Total undiscounted life years†	3.321	3.368	3.437	−0.047	−0.116
Life years*: Pre-progressed	0.876	0.675	0.594	0.201	0.282
Life years*: Post-progressed	2.167	2.406	2.546	−0.240	−0.379
Total discounted life years*	3.043	3.082	3.140	−0.039	−0.097
Incremental cost per QALY				Dominant	Dominant
Incremental cost per LY				Less effective	Dominant
*discounted †undiscounted					

*EVE: everolimus; EXE: exemestane; BEV: bevacizumab; PACL: paclitaxel; CAPE: capecitabine; QALY: quality-adjusted-life years; mBC: metastatic breast cancer; LY: life year*

### Sensitivity analysis

In addition, sensitivity analyses of the model were performed, regarding the method used to extrapolate OS and PFS data. The choice of parametric distribution was found to have an effect on the main findings of our study, but only in terms of the comparison between EVE plus EXE and BEV plus CAPE. To be more precise, EVE plus EXE was a dominant option over BEV plus PACL, regardless of the parametric distribution used to extrapolate OS and PFS, but it was deemed less effective with respect to BEV plus CAPE, when the exponential (endpoint), Gompertz or exponential (curve) distributions were applied (Table 3).

**Table 3:** Results from the sensitivity analysis

Base case ICER	Dominant	Low value		High Value	
Parameter	Base case value	High value	ICER	Low value	ICER
PFS: EVE plus EXE vs. BEV plus PACL (−50 %; +50 %)		50 %	1,384 €	150 %	Dominant
PFS: BEV plus PACL (−50 %; +50 %)		50 %	Dominant	150 %	Dominant
PFS: EVE plus EXE vs. BEV plus CAPE (−50 %; +50 %)		50 %	26,091 €	150 %	Dominant
PFS: BEV plus CAPE (−50 %; +50 %)		50 %	Dominant	150 %	Dominant
OS: EVE plus EXE vs. BEV plus PACL (−50 %; +50 %)		50 %	€11,251 €	150 %	Dominant
OS: BEV plus PACL (−50 %; +50 %)		50 %	Dominant	150 %	Dominant
OS: EVE plus EXE vs. BEV plus CAPE (−50 %; +50 %)		50 %	17,104 €	150 %	Dominant
OS: BEV plus CAPE (−50 %; +50 %)		50 %	Dominant	150 %	5,876 €
Fixed post-progression survival (6–48 months)	12	6	Dominant	48	Dominant
Utility: pre-progression (0.36; 0.90)	0.773	0.36	Dominant	0.90	Dominant
Utility: post-progression (0.2; 0.97)	0.496	0.22	Dominant	0.97	Dominant
Pre-progression background costs (50 €; 150 €)	95.16 €	50.00 €	Dominant	150 €	Dominant
Post-progression background costs (500 €; 1500 €)	1,057.41 €	500.00 €	Dominant	1,500 €	Dominant
Adverse event costs: EVE plus EXE (38 €; 133 €)	62 €	37.89 €	Dominant	133 €	Dominant
Adverse event costs: BEV plus PACL (4.92 €; 34 €)	5.14 €	4.92 €	Dominant	34 €	Dominant
Adverse event costs: BEV plus CAPE (0.50 €; 34 €)	1 €	0.50 €	Dominant	34 €	Dominant
Adverse event disutilities: EVE plus EXE (0.011; 0.04)	−0.029	−0.011	Dominant	−0.04	Dominant
Adverse event disutilities: BEV plus PACL (0.005; 0.069)	0.027	−0.005	Dominant	−0.069	Dominant
Adverse event disutilities: BEV plus CAPE (0.005; 0.069)	−0.0031	−0.002	Dominant	−0.069	Dominant
Adverse events: unknown disutility assumption (−0.01; −0.10)	−0.05	−0.013	Dominant	−0.100	Dominant
BOLERO II Central PFS included	Without			With	Dominant
Fixed post-progression survival applied	Without			With	Dominant
Dose intensity included	Without			With	Dominant
Drug cost: EVE plus EXE vs. BEV plus PACL (2000 €; 5000 €)	2,447.75 €	2000 €	Dominant	5,000 €	324,159 €
Drug cost: BEV plus PACL (2000 €; 5000 €)	3,806.42 €	2000 €	151,243 €	5,000 €	Dominant
Drug cost: EVE plus EXE vs. BEV plus CAPE (2000 €; 5000 €)	2,447.75	2000 €	Dominant	5,000 €	4,082,144 €
Drug cost: BEV plus CAPE (2000 €; 5000 €)	3,510.97€	2000 €	3,539,497 €	5,000 €	Dominant
Extrapolation method (EVE plus EXE versus comparator)					
Exponential, *endpoint* (PACL)	Dominant				
Exponential, *endpoint* (CAPE)	Less effective				
Gompertz (PACL)	Dominant				
Gompertz (CAPE)	Less effective				
Exponential, *curve* (PACL)	Dominant				
Exponential, *curve* (CAPE)	Less effective				
Log-logistic(PACL)	Dominant				
Log-logistic (CAPE)	Dominant				

*EVE: everolimus; EXE: exemestane; BEV: bevacizumab; PACL: paclitaxel; CAPE: capecitabine; ICER: incremental cost effectiveness ratio; PFS: progression free survival; OS* **:** *overall survival*

Finally, the one-way sensitivity analysis revealed that the results of our study were mainly driven by the PFS and OS data as well as the drug acquisition costs. In this context, EVE plus EXE was not a dominant alternative, but remained cost-effective over BEV plus PACL when the PFS of EVE plus EXE was reduced by 50 % (ICER: €1,384). Likewise, when the OS of EVE plus EXE was decreased by 50 % the resulting ICER reached €11,251 – well below the predetermined WTP threshold of €36,000 per QALY gained. Similarly, in the comparison of EVE plus EXE over BEV plus CAPE, the former was a cost-effective alternative when its PFS and OS were reduced by 50 % (ICER: €26,091 and ICER: €17,104, respectively) and when the OS of BEV plus CAPE was increased by 50 % (ICER: €5,876). All these results are presented in Table 3.

### Probabilistic sensitivity analysis

**Fig. 4:**
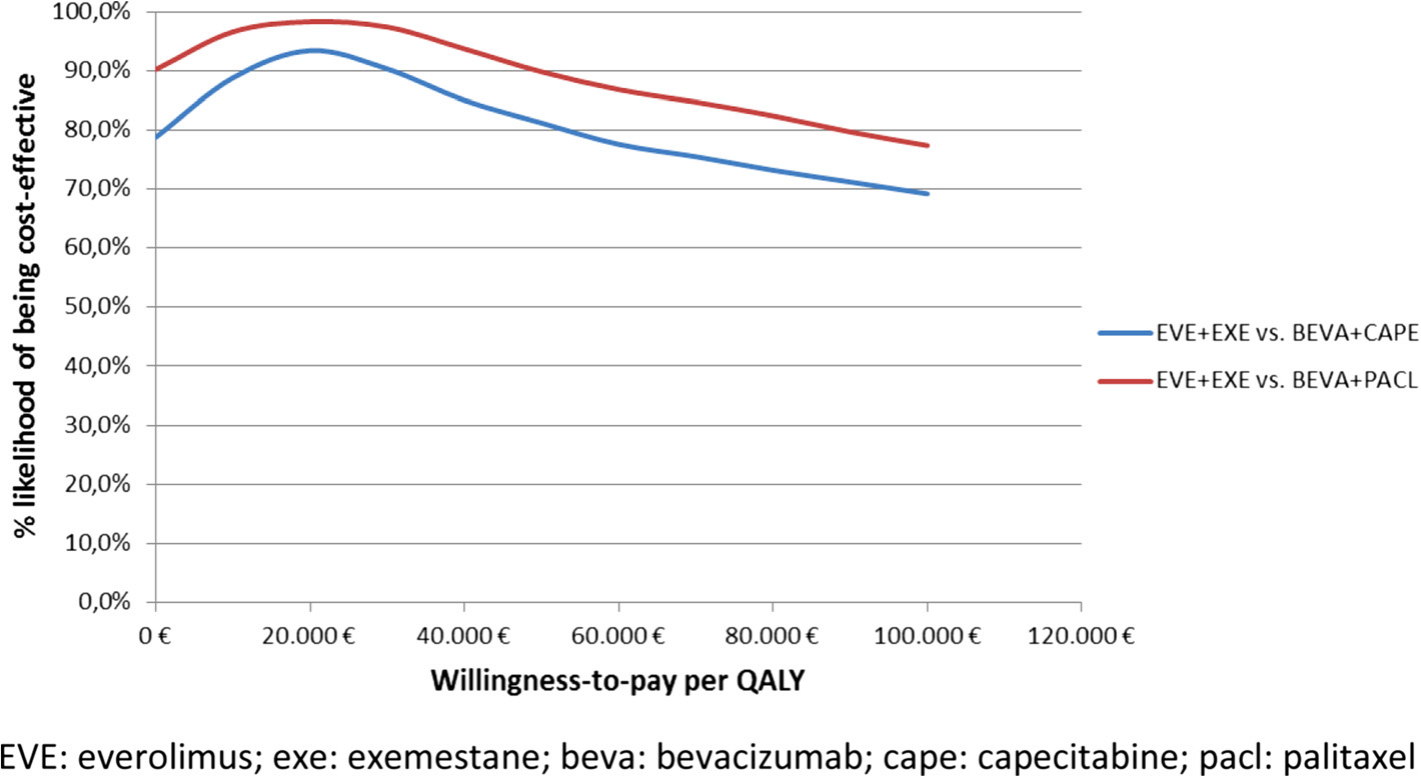
Cost-Effectiveness Acceptability Curves

The PSA confirmed the deterministic results (Fig. 4). The CEAC showed that EVE plus EXE was cost-effective over BEV plus PACL in 95.5 % of cases and over BEV plus CAPE in 87.2 %, at a WTP of €36,000.

## Discussion

In the present study, a Markov model was adapted to assess the cost-effectiveness of EVE plus EXE against BEV plus CAPE and BEV plus PACL for the treatment of ER+/HER2- advanced BC in the Greek health care setting. The parameters considered in the Markov model were obtained from the literature and a local expert. According to the base case results, EVE plus EXE dominates both active comparators, as it is associated with lower costs and higher clinical efficacy in both cases.

At this point, it should be highlighted that this is the first study to evaluate the cost-effectiveness of EVE plus EXE over chemotherapy plus BEV in postmenopausal women with ER + BC. The manufacturer of EVE had submitted an economic evaluation of EVE plus EXE for treating HR+/HER2- advanced BC after endocrine therapy for review by the NICE. The comparators considered in this previous analysis by the manufacturer were EXE alone, TAM, FULV, DOC, doxorubicin (DOX), and CAPE. According to the base case results of the manufacturer, EVE plus EXE compared to EXE alone generated incremental costs of £27,086, as well as 0.84 incremental QALYs (ICER: £32,417/QALY). Compared to TAM, EVE plus EXE had an ICER of £29,109 per QALY gained. Regarding the comparison with FULV, the EVE plus EXE arm resulted in additional costs of £20,937 and 0.77 incremental QALYs (£27,147/QALY). For the comparison with DOC, EVE plus EXE provided an ICER of £11,000 per QALY gained (£13,364 incremental costs and 1.21 incremental QALYs). EVE plus EXE versus DOX had an ICER of £20,253 per QALY gained, while with respect to CAPE, EVE plus EXE was more costly by £29,597 and more effective by 1.21 QALYs (ICER: £24,362/QALY). Nonetheless, based on the Evidence Review Group analysis that used a non-parallel exponential model and local PFS measurements, the EVE plus EXE arm versus EXE was not cost-effective, as it provided an ICER of £68,000 per QALY gained.

The analysis pursued was characterized by specific drawbacks and limitations. First of all, limitations in the model arise from the nature of the underlying data, which in several cases were not available with the required level of detail. In order to overcome this impediment, conservative assumptions and indirect comparisons were made (i.e. similar clinical efficacy for chemotherapy agents) that could raise issues of structural uncertainty Nonetheless, indirect comparison is considered as a valid method as long as large direct comparative trials are lacking. In particular, the approach of indirect comparison is possible in cases where trials are consistent in terms of the outcomes used as endpoints, the way these outcomes are reported, the baseline population, the dosages of medication, and the follow-up time. Another limitation of the study was the assumption that the post-progressed treatment sequences did not affect survival or utility but only the costs arising; nonetheless, this was a structural and essential assumption in the core model due to the lack of data on the efficacy of these treatment sequences. This assumption was considered valid in the core model, as the benefit should already have been accounted for in the OS data from the clinical trials. In addition, in the absence of mBC patient registries in Greece, the methodology to collect resource use data may be susceptible to bias (specialized key opinion leader [KOL]) but it still represents one of the recommended methods for collecting resource utilization data. Finally, it should be noted that the results have to be considered strictly in the Greek setting and on the basis of the present time resource and drug prices. If any of the underlying parameters change, so may the results and the conclusions of the analysis, resulting in limited external validity of the model and the outcomes. Nonetheless, a series of sensitivity analyses indicated that our model and outcomes are valid, since the main results remained unchanged.

## Conclusions

Using conservative assumptions, the present economic evaluation suggests that EVE plus EXE provides greater adjusted survival and is less costly compared to BEV plus PACL and BEV plus CAPE. Therefore, EVE plus EXE should be considered as a cost-saving intervention in Greek health care for the management of postmenopausal women with HR+/HER2- advanced BC progressing on NSAI.

## Additional files

Additional file 1: Appendix I. Resource consumption during the pre-progressed stage. (DOCX 72 kb) Additional file 2: Appendix II. Unit costs considered in the model. (DOCX 73 kb) Additional file 3: Appendix III. Resource consumption during the post-progressed stage (the same post-progression management was assumed for all comparators). (DOCX 69 kb)
