# Supplementary material for: Everolimus plus exemestane versus bevacizumab-based chemotherapy for second-line treatment of hormone receptor-positive metastatic breast cancer in Greece: An economic evaluation study
Source: BMC Health Serv Res. 2015 Aug 5;15:307. doi: 10.1186/s12913-015-0971-4 (PMC4524048; doi:10.1186/s12913-015-0971-4)
Supplement: Additional file 1: Appendix I. — Resource consumption during the pre-progressed stage. (DOCX 72 kb) [file 12913_2015_971_MOESM1_ESM.docx]

**APPENDIX I: Resource consumption during the pre-progressed stage**

| **Resource use** | **EVE plus EXE** | | **BEV plus PACL** | | **BEV plus CAPE** | |
| --- | --- | --- | --- | --- | --- | --- |
| **Drug Dose** | EVE: 10 mg/day  EXE: 25 mg/day | | BEV: 10 mg/kg on days 1 & 15  PACL: 90 mg/m^2^ on days 1, 8 & 15 | | BEV: 15 mg/kg every 3 weeks  CAP: 1000 mg/m^2^ twice daily on days 1 to 14 | |
| **Number of IV administrations** | 0 | | 3 per 28 days | | 1 per 21 days | |
| **Pre-treatment resources** | **% of utilization** | **Number of units** | **% of utilization** | **Number of units** | **% of utilization** | **Number of units** |
| Dexamesthasone  (8 mg twice daily for 3 days) | 0% | - | 100% | 3 times/28 days | 100% | 1 time/21 days |
| Antihistamine  (1 pill twice daily for 3 days) | 0% | - | 100% | 3 times/28 days | 100% | 1 time/21 days |
| Ondansetron 8 mg  (16 mg daily for 3 days) | 0% | - | 100% | 3 times/28 days | 100% | 1 time/21 days |
| **Prophylactic treatment** |  |  |  |  |  |  |
| Pegfilgrastim | - | - | - | - | 20% | 1/21 days |
| Filgrastim | - | - | 40% | 5/28 days | 20% | 1/21 days |
| Epoetin (150 mg) | - | - | 20% | 1/week | 20% | 1/week |
| Darbepoetin (150 mg) | - | - | 20% | 1/week | 20% | 1/ week |
| **Monitoring resources** |  |  |  |  |  |  |
| Complete blood count | 100% | 1 per month | 100% | 3/month | 100% | 2/month |
| Liver function tests | 100% | 1 per month | 100% | 1/month | 100% | 1/month |
| General urine test | 0% | - | 100% | 2/28 days | 100% | 1/21 days |
| CT scan | 50% | 1 per 3 months | 50% | 1/3 months | 50% | 1/3 months |
| MRI | 50% | 1 per 3 months | 50% | 1/3 months | 50% | 1/3 months |
| Bone scan | 20% | 1 annually | 20% | 1/year | 20% | 1/year |
| **Adverse events management** | **% of episodes require hospitalization** | | **Length of hospitalization** | | **Resource consumption in outpatient setting** | |
| Nausea |  | |  | | Ondansetron (16 mg daily for 3 days) | |
| Penumonitis | 100% | | 7-10 days | | IV antibiotics | |
| Allergy |  | |  | | Dexamethasone (20 cc twice daily for 3 days) | |
| Anaemia |  | |  | | Darbepoetin (150 mg) once weekly weekly until Hb> 9 / 5of patients require blood transfusion | |
| Neutropenia |  | |  | | Filgrastim 1 daily for 3 days | |
| Arthralgia | 5% | | 3 days | | Dexamethasone (8 mg twice daily for 5 days) | |
| Myalgia | 5% | | 3 days | | Dexamethasone (8 mg twice daily for 5 days) | |
| Vomiting |  | |  | | Ondansetron (16 mg daily for 3 days) | |
