# Supplementary material for: Everolimus plus exemestane versus bevacizumab-based chemotherapy for second-line treatment of hormone receptor-positive metastatic breast cancer in Greece: An economic evaluation study
Source: BMC Health Serv Res. 2015 Aug 5;15:307. doi: 10.1186/s12913-015-0971-4 (PMC4524048; doi:10.1186/s12913-015-0971-4)
Supplement: Additional file 2: Appendix II. — Unit costs considered in the model. (DOCX 73 kb) [file 12913_2015_971_MOESM2_ESM.docx]

**APPENDIX II: Unit costs considered in the model**

| **Description** | |  | **Source** | |
| --- | --- | --- | --- | --- |
| **Model comparators** | | **Cost per dose** |  | |
| EVE plus EXE  EVE  EXE | |  | Price bulletin, August 2013, Ministry of Health | |
|  |  | 91.36 € |  |  |
|  |  | 1.85 € |  |  |
| BEV plus PACL  BEV  PACL | |  | Price bulletin, August 2013, Ministry of Health | |
|  |  | 1,679.94 € |  |  |
|  |  | 237.25 € |  |  |
| BEV plus CAPE  BEV  CAPE | |  | Price bulletin, August 2013, Ministry of Health | |
|  |  | 2,519.91 € |  |  |
|  |  | 7.28 € |  |  |
| **Premedication Cost** | |  |  | |
| Dexamesthasone | | 1.81 € |  | |
| Antihistamine | | 0.165 € | Price bulletin, August 2013, Ministry of Health | |
| Ondansetron | | 4.49 € |  | |
| **Prophylactic Treatment** | |  |  | |
| Pegfilgrastim | | 658.41 € | Price bulletin, August 2013, Ministry of Health | |
| Figrastim | | 25.90 € |  |  |
| Epoetin | | 74.84 € |  |  |
| Darbepoetin | | 176.29 € |  |  |
| **Post-progression state drugs** | |  |  | |
| Fulvestrant | | 550.02 € | Price bulletin, August 2013, Ministry of Health | |
| Capecitabine + Navelbine | | 65,08 € |  |  |
| Docetaxel | | 317.49 € |  |  |
| *Fendanyl paches*  *Lonarid* | | 6.08 € |  |  |
|  |  | 0.13 € |  |  |
| **Drug administration** | |  |  | |
| Cost / IV Administration | | 80 € | Ministerial Decision 104494, 26/9/2011. This is the cost reimbursed by EOPPY for day case treatment. | |
| **Monitoring Resources** | **Cost per unit** | | |  |
| Complete blood count | | 2.88 € | Ministerial Decision (FEK B’ 3054/18-11-2012, 49976/05-12-2012, 3100/2011) | |
| Liver function tests | | 27.32 € |  |  |
| Kidney function Test | | 6.31 € |  |  |
| General urine test | | 1.76 € |  |  |
| Glucose | | 2.26 € |  |  |
| Calcium | | 4.05 € |  |  |
| CT scan | | 71.10 € |  |  |
| MRI | | 236.95€ |  |  |
| Bone scan | | 60.00 € |  |  |
| **Adverse event Management Cost** | | **One-off costs** |  | |
| Nausea | | 13.47 € | 1. Cost per inpatient day: 60€ (FEK 3054/18-11-2012).  2. Outpatient Setting: The cost assigned was calculated based on resources used such as physicians’ visits and medication. Resource consumption and utilization were based on expert’s opinion and were combined with the corresponding unit costs obtained from government gazzette and the drug price bulletin | |
| Penumonitis | | 540 € |  |  |
| Allergy | | 18.10 € |  |  |
| Anaemia | | 766.82 € |  |  |
| Neutropenia | | 77.71 € |  |  |
| Arthralgia | | 27.10 € |  |  |
| Myalgia | | 27.10 € |  |  |
| Vomiting | | 13.47 € |  |  |
| **End-of-life cost (one-off)** | | | | |
| 1 month | | 823.60 € | Same resource use per month as in the supportive palliative care line | |
