# Supplementary material for: Everolimus plus exemestane versus bevacizumab-based chemotherapy for second-line treatment of hormone receptor-positive metastatic breast cancer in Greece: An economic evaluation study
Source: BMC Health Serv Res. 2015 Aug 5;15:307. doi: 10.1186/s12913-015-0971-4 (PMC4524048; doi:10.1186/s12913-015-0971-4)
Supplement: Additional file 3: Appendix III. — Resource consumption during the post-progressed stage (the same post-progression management was assumed for all comparators). (DOCX 69 kb) [file 12913_2015_971_MOESM3_ESM.docx]

**APPENDIX III: Resource consumption during the post-progressed stage (the same post-progression management was assumed for all comparators)**

| **Post progression treatment sequence** | **% patients** | **Dosing Schedules** | **Treatment duration** | **Length of Stay** | **Monitoring resources** |
| --- | --- | --- | --- | --- | --- |
| **3rd line hormonal therapy, 4th line chemotherapy, supportive palliative care** | 50% |  |  |  |  |
| 3^rd^ line hormonal therapy |  | Fulvestrant 500 mg:  4 injections in the 1^st^ month and 1 injection/month for subsequent months | 10-12 months | 0 days | Complete blood count: 1/month  Liver function test: 1/month  CT scan: 1/6 months |
| 4^th^ line chemotherapy |  | Taxotere 80 mg  (2 vials/21 days) | 6 months | 5 days/month | Complete blood count: 2/ 21 days  Liver function test: 1/21 days  CT scan: 1/3 months |
| Supportive palliative care |  | Lonarid: 1 tablet x 3/day  Fentanyl paches: 1 per 3 days | 6 months | 10-15 days/month | - |
| **3^rd^ and 4^th^ line chemotherapy, supportive palliative care** | 50% |  |  |  |  |
| 3^rd^ line chemotherapy |  | Xeloda 500 mg: 7 tabs/day  for 14 days (21-day cycle)  Navelbine 30 mg: 1 cap x 3  on days 1 and 8 (21-day cycle) | 6 months | 5 days/month | Complete blood count: 1/month  Liver function test: 1/month  CT scan: 1/3 months |
| 4^th^ line chemotherapy |  | Taxotere 80 mg  (2 vials every 21 days) | 6 months | 5 days/month | Complete blood count: 2/21 days Liver function test: 1/21 days  CT scan: 1/3 months |
| Supportive palliative care |  | Lonarid: 1 tablet x 3/day  Fentanyl patches: 1/3 days | 6 months | 10-15 days/month | - |
